# Enterohemorrhagic *E. coli* (EHEC) and the microbiome

**DOI:** 10.1371/journal.ppat.1013224

**Published:** 2025-06-12

**Authors:** Quentin Perraud, Vanessa Sperandio

**Affiliations:** 1 Department of Medical Microbiology and Immunology, School of Medicine and Public Health, University of Wisconsin-Madison, Madison, Wisconsin, United States of America; Duke University School of Medicine, UNITED STATES OF AMERICA

## EHEC

Enterohemorrhagic *E. coli* (EHEC) is a food-borne pathogen that infect the human lower intestine and causes hemorrhagic colitis, which can in some cases lead to hemolytic uremic syndrome (HUS), a life-threatening condition. Most bacterial pathogens historically responsible for deadly epidemics have become a thing of the past in the developed world through progress in sanitation and public health policies, however food-borne bacterial pathogens still represent a significant cause of morbidity and mortality. EHEC is no exception to this: the CDC estimates that 265,000 EHEC infections occur each year in the United States.

While most bacterial infections can still be managed through the use of antibiotics, EHEC represents a unique case where the use of antibiotics is not recommended. This is due to the phage-encoded Shiga-toxin present in EHEC’s genome, a virulence factor directly responsible for the development of HUS [1]: it has been shown that antibiotic treatment results in an increase of production of Shiga-toxin [2]. This lack of therapeutic solutions has led to EHEC being an important subject for research focused on novel, non-antibiotic-based methods to manage the disease [3,4], mainly based on targeting EHEC’s many virulence factors. Despite this focus on EHEC and its pathogenesis, the lack of an animal model perfectly recapitulating the disease caused in humans means that studying host-pathogen interactions remain challenging.

**Fig 1:**
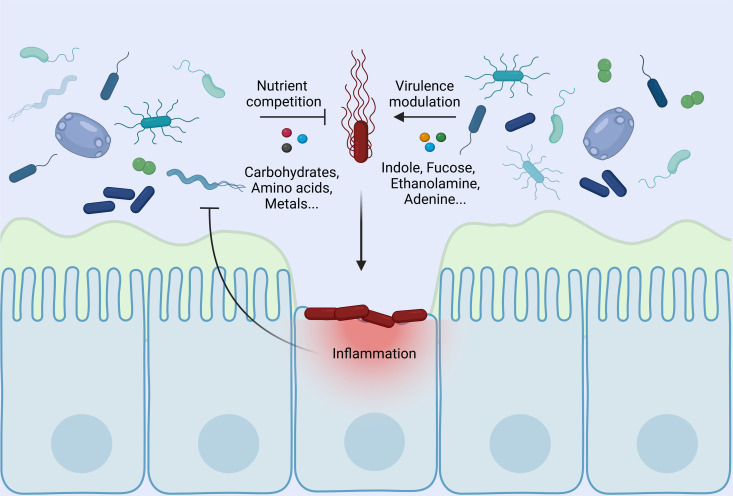
Native species of the gut microbiome interact with EHEC by outcompeting the pathogen for crucial nutrients. However, these microbes also release chemical cues that trigger EHEC’s virulence program, promoting its attachment to the colonic epithelium. The inflammation caused by EHEC alters the metabolic landscape within the colon lumen, disrupting the conditions needed for strictly anaerobic commensals to thrive and alleviating some of the competitive pressure on the pathogen. Figure created with biorender.com.

One of the many enigmas surrounding EHEC’s pathogenesis is the impact of the host microbiome on the infection. Numerous studies involving gnotobiotic animal models clearly identify the microbiome as a key factor in maintaining homeostasis and preventing disease, and the advent of high-throughput sequencing and metabolomic approaches now give rise to the opportunity to better characterize these effects through a more holistic and mechanistic lens. In the case of EHEC, the traditional view of the gut microbiome as having a protecting role against intestinal colonization is also now nuanced by the fact that it produces a variety of signal molecules exploited as cues by EHEC to regulate its virulence program (Fig 1), and might be involved in Shiga-toxin driven disease through the conversion of the commensal microbiome by the Shiga-toxin encoding phage [5].

### The microbiome as a barrier to EHEC colonization

The importance of the host microbiome in EHEC infection can be inferred from some of the animal models used to study EHEC infection: the infant rabbit model, the antibiotic-treated mouse and the gnotobiotic pig models. The streptomycin-treated mouse model has been used extensively to study the mechanism of action of Shiga-toxin [6] or *in vivo* nutrient requirement of EHEC [7] due to its relative simplicity and the genetical tractability of the murine host. This model came to be used due to EHEC’s inability to thrive in a mouse with an intact microbiota. It involves a very high bacterial inoculum and the depletion of resident anaerobes in order to increase the odds of EHEC establishing itself in the mouse gut. However, it is notable that EHEC does not cause intestinal disease in these animals. The infant rabbit model also underscores the role of the microbiota as a resistance barrier to colonization: infant animals have a poorly diverse and non-mature microbiota, slowly developing during the first weeks of their lives. The gnotobiotic pig model is germ-free. The susceptibility of germ-free animals to EHEC is not that surprising considering that germ free animals will readily get colonized by microbes isolated from very diverse sources [8]. The common denominator of all those animal models of EHEC infection is that they require the absence or alteration of the microbiota. The murine pathogen *Citrobacter rodentium* is often used by the field to bypass this limitation and extrapolate a closer scenario to EHEC infections. *C. rodentium* pathogenesis is also dependent on the LEE (locus of enterocyte effacement) pathogenicity island [9].

This horizontally transferred genomic region encodes for a type III secretion system (TIIISS) and its regulator (*ler*), secreted effectors, the adhesin intimin (*eae*) and the intimin receptor (*tir*) that is translocated into the host cell through the TIIISS, allowing strong attachment [10,11]. This island is essential for EHEC and *C. rodentium* pathogenesis. Genetic manipulation of *C. rodentium* led to the introduction of the lambdoid phage that encodes Shiga-toxin, better recapitulating features of EHEC-mediated infections [12]. Just as with the other models previously described, the gut microbiome also induces colonization resistance. In the case of *C. rodentium*, it has been shown that antibiotic treated mice are a more susceptible to infection.

While these models underscore a protective effect for the gut microbiome when the host is exposed to EHEC, the actual mechanisms of this colonization resistance have proven difficult to pinpoint. Various hypotheses have been advanced as a basis for this resistance: competition for space and nutrient between species, competition through inhibition/killing and indirect competition through “training” of the immune system provided by a healthy microbiome.

### Mechanism of interspecies competition in the microbiota

#### Nutrient competition.

The earliest explanation of this protective effect of the gut microbiome against invading pathogens is Rolf Freter’s nutrient-niche hypothesis [13], in which the gut is compared to a bioreactor where species exist in an equilibrium determined by the availability of nutrients and an invading species needs to adapt to use a limiting nutrient better than its competitors to colonize this ecosystem. Accordingly, it has been shown that EHEC uses a small set of carbon sources in the mouse intestine, and pre-colonizing a mouse with commensal strains using the same subset of carbon sources leads to colonization resistance [14]. It has also been shown that the ability to use galacturonic acid and glucuronic acid, nutrients that are not exploited by commensal *E. coli*, are important for colonization [15,16]. Forty years of experimental data have supported Freter’s hypothesis to a certain degree, while refining it to newer models such as Conway and Cohen’s restaurant hypothesis [17], taking into account spatiotemporal heterogeneity in the gut and postulating the existence of a variety of “restaurants” defined by a very localized availability of nutrients due to the physical proximity of a bacterium with fiber and/or mucus degrading commensals. While most studies of nutrient-driven colonization resistance have been focused on carbon source utilization, competitions for other nutrients have also been shown to be important for colonization. A recent study showed that *C. rodentium* colonization of the mouse gut is driven by an induction of amino acid biosynthesis pathways, bypassing nutrient competition with commensals in an infection context [18]. Some microbiota-derived molecules also moonlight as a nutrient source and signaling molecule for EHEC, for instance ethanolamine released by the commensal gut microbiome and the host, has been shown to be used as both a source of nitrogen and a signal that activates expression of the LEE pathogenicity island [19].

Another molecule that is not classically thought of as a nutrient but provides a way for facultative anaerobes enteric pathogens to compete with the resident microbiome is oxygen. The colitis caused by enteric pathogens can lead to oxygenation of the environment, depleting the strictly anaerobic commensals to alleviate nutritional competition [20].

#### Antibacterial compounds.

Another mechanism underscoring microbiome-mediated colonization resistance by EHEC is the production of antimicrobial peptides, a common defense strategy for the resident gut microbiota. Genera whose members are known to produce bacteriocins are *Lactobacillus* (plantaricins and lactocins), *Bacteroides* (bacteroidetocins), *Enteroccocus* (enterocins) and even commensal *Escherichia* (colicins, microcins) [21]. These small ribosomal synthesized peptides present a variety of mechanisms of action, such as disrupting cell wall and membranes or targeting intracellular processes.

#### Mucus production.

The gut epithelium is protected by a mucus barrier presenting an outer layer of loose mucus colonized by commensals and an inner layer, which is denser and impermeable to bacteria. This protective barrier is affected by resident members of the microbiome: *Bacteroides*, *Faecalibacterium* [22], *Lactobacillus* [23], and *Akkermansia muciniphila* [24] are known to stimulate mucus production through the production of metabolites such as short chain fatty acids (acetate, propionate, butyrate) that are recognized by the host cells leading to an increase in mucus production. This mucus layer can then be enzymatically degraded and utilized as carbon sources by the microbiome.

### The microbiome as a source of signaling molecules

**Fig 2:**
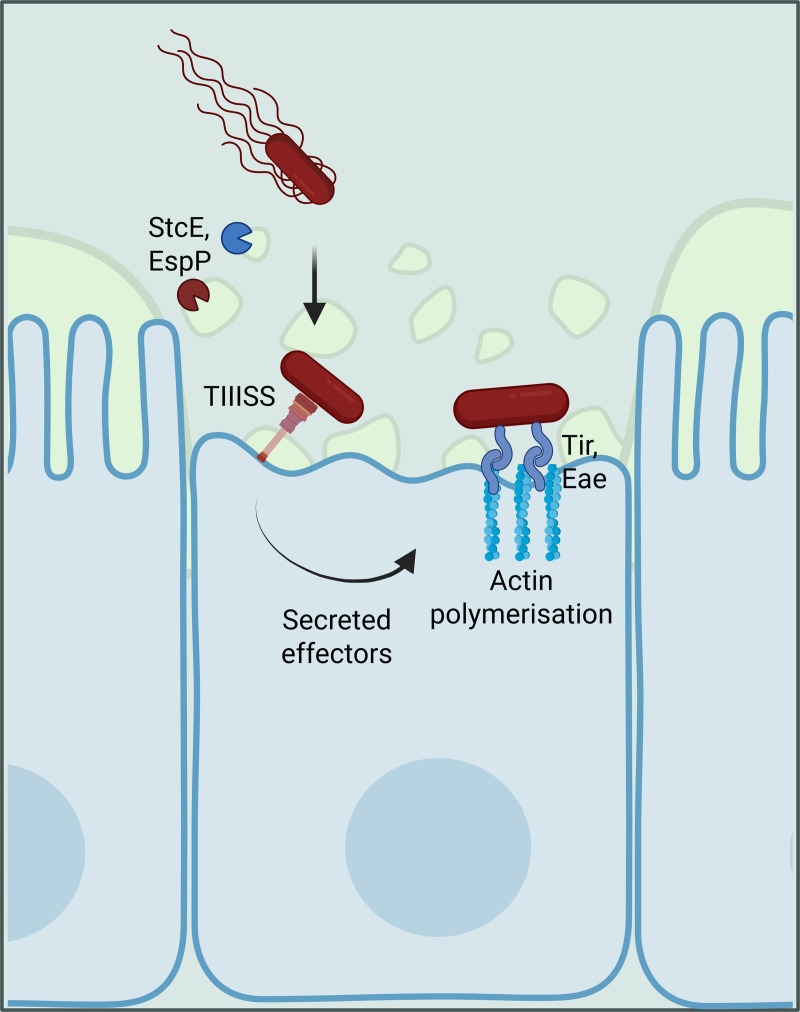
EHEC degrades the mucus layer forming a physical barrier between the colon’s lumen and the epithelium through the action of the StcE mucinase and the EspP protease, allowing direct contact between bacteria and epithelial cells. This contact leads to injections of effectors through the bacterial type three secretion system and strong attachment to the epithelium chiefly involving the Tir/Eae adhesin/receptor protein pair. This adhesion mechanism is supplemented by a wide range of other adhesins reviewed in McWilliams and Torres [27]. Figure created with biorender.com.

Within the host EHEC senses several chemical cues to reach the colon epithelium where it can attach and thrive. First it disrupts the mucus layer, a step facilitated by the plasmid-encoded StcE mucinase [25] and the EspP protease [26], as well as by adhesins binding to glycans. Once in contact with the epithelium, the LEE-encoded type III secretion system injects effectors into the host cell, leading to a strong attachment between the host cell and the pathogen through the Tir and Eae proteins previously described (Fig 2). This allows EHEC to bypass the normal requirement for a gut resident to multiply fast enough to overcome the rate of gut transit. This complex machinery has been the subject of intense scrutiny ever since its discovery, due to its essential role in the bacteria’s ability to cause disease.

The expression of the LEE genes is tightly regulated by a variety of microbiome-derived molecular cues, allowing the bacteria to only express its virulence program once it reaches its niche within the lower intestine.

Perhaps the most studied example of interactions with a commensal bacterium shaping EHEC’s colonization and virulence involve *Bacteroides thetaiotaomicron*. As previously discussed, *B. thetaiotaomicron* interacts with the mucus layer by stimulating mucus production and degrading it, using mucus building blocks as carbon sources. Fucose, one of these building blocks, can be recognized by EHEC through a two-component system composed of the histidine kinase FusK and the associated response-regulator FusR. Two-component systems are signal transduction pathways allowing the regulation of gene expression in response to environmental cues. The *fusKR* genes are encoded within a pathogenicity island not found in commensal *E. coli* and Enterobacteriaceae. It has been shown that this sensing leads to the modulation of the LEE pathogenicity island expression, increasing EHEC’s ability to cause disease in an infant rabbit model [28]. The same kind of study has been carried out with other enteric pathogens, elegantly showing that despite its role as a barrier to colonization, the microbiome and its associated metabolome can itself lead to increase in pathogenicity [29,30]. A more recent example of EHEC-commensal interaction shaping virulence can be found in EHEC’s interaction with *Enterococcus faecalis*: it has been shown that *E. faecalis* derived adenine can be taken up and used as a molecular cue by EHEC, leading to an increase of LEE expression [31]. Another example of microbiota-derived metabolite strongly impacting EHEC’s ability to colonize can be found in the sensing of indole by EHEC. It has been shown that indole is directly sensed by EHEC through the CpxAR two component system, repressing the expression of the LEE pathogenicity island [32]. It is thought that this sensing of a metabolite mostly present in the lumen of the gut allows EHEC to not waste energy producing its type three secretion apparatus until it is closer to the epithelial barrier.

As we already previously described for the case of ethanolamine, some metabolites blur the line between nutrient and signaling molecules, and a growing body of work dissecting virulence regulation in EHEC displays the ingenious ways evolution worked toward interconnecting all aspects of bacterial physiology with virulence in the case of EHEC [33].

### Forgotten members of the microbiome

The main focus of most microbiome studies remains on bacteria even though archaea (ex: *Methanobrevibacter smithii, Methanosphaera stadtmanae*) [34] and fungi (ex: *Candida albicans*) [35] are also recognized as important components of the microbiome. The paucity of the literature concerning interspecies and interkingdom interactions between these members of the gut microbiota can be explained by a combination of technical and biological factors (difficulty in isolation, cultivation and sequencing). While physical proximity does not necessarily equate to complex interactions, a few studies hint at a potential role of those poorly characterized members of the human microbiome in the invasion of gut pathogens. It has been shown that *Salmonella* is able to directly interact with *C. albicans* through its type III secretion system to cause the release of arginine [36], an amino acid that directly regulates the virulence program of EHEC [37], leading to the question of whether or not other enteric pathogens could make use of resources produced by fungi. Another one of those fungi–*Salmonella* interactions is the use of fungal siderophore by *Salmonella* to promote colonization in the mouse gut by facilitating the uptake of iron [38], through a siderophore-specific uptake system also present but poorly characterized in EHEC.

### Perspectives

Since it was first isolated in 1982 [39], EHEC has been recognized as a major cause of morbidity worldwide and has been the subject of a lot of research to better understand its pathogenesis [40]. However, a lot of questions are left to be answered concerning its interaction with the host microbiome, how it establishes its niche and its ability to colonize humans. The course and severity of these infections can vary from almost asymptomatic to life threatening. Differences in immunological responses can be at play, but the characteristics defining these more severe outcomes might also lie in the composition of the host microbiome at the moment of exposure.

These questions might prove difficult to solve, due to the lack of an animal model that perfectly recapitulates EHEC’s pathogenesis, and temporal and inter-individual diversity in the human microbiome. However, these questions are important and quite crucial at a time when the increase of industrialization of livestock husbandry in the developing world might lead to more frequent epidemics.

The intestine is perhaps the most studied organ when it comes to microbiome composition, due to it being the main reservoir of microorganisms in our bodies but studying the composition of the microbiome of other parts of the gastrointestinal tract might also lead to insights into how EHEC is able to recognize its niche. The main focus of EHEC-microbiome interaction studies has so far been in the intestine, however EHEC also encounters the other compartments of the gastrointestinal tract during its infectious cycle, which we know harbor microbiota in their own right. An exciting new avenue of research would be evaluating whether or not the transient interaction between EHEC and these commensals is going to affect colonization.

## Abbreviations

EHEC: enterohemorrhagic *E. coli*
HUS: hemolytic uremic syndrome
LEE: locus of enterocyte effacement
TIIISS: type III secretion system
